# Inhibiting Anti-angiogenic VEGF165b Activates a Novel miR-17-20a-Calcipressin-3 Pathway that Revascularizes Ischemic Muscle in Peripheral Artery Disease

**DOI:** 10.21203/rs.3.rs-3213504/v1

**Published:** 2023-08-14

**Authors:** S Batan, S Kuppuswamy, M Wood, M Reddy, BH Annex, VC Ganta

**Affiliations:** 1 Vascular Biology Center, Department of Medicine, Augusta University, Augusta-GA-30912; 2 Medical College of Georgia, Augusta University, Augusta-GA-30912

**Keywords:** OncomiR, Ischemia, Angiogenesis, Macrophage polarization, VEGF-A isoforms

## Abstract

**Background:**

VEGF_165_a increases the expression of microRNA-17-92 cluster, promoting developmental, retinal, and tumor angiogenesis. We have previously shown that VEGF_165_b, an alternatively spliced VEGF-A isoform, inhibits the VEGFR-STAT3 pathway in ischemic endothelial cells (ECs) to decrease their angiogenic capacity. In ischemic macrophages (Møs), VEGF_165_b inhibits VEGFR1 to induce S100A8/A9 expression, which drives M1-like polarization. Our current study aims to determine whether VEGF_165_b inhibition promotes perfusion recovery by regulating the miR-17-92 cluster in preclinical PAD.

**Methods:**

Hind limb ischemia (HLI) induced by femoral artery ligation and resection was used as a preclinical PAD model. Hypoxia serum starvation (HSS) was used as an *in vitro* PAD model. VEGF_165_b was inhibited/neutralized by an isoform-specific VEGF_165_b antibody.

**Results:**

Systematic analysis of miR-17-92 cluster members (miR-17-18a-19a-19b-20a-92) in experimental-PAD models showed that VEGF_165_b-inhibition induces miRNA-17-20a (within miR-17-92 cluster) in HSS-ECs and HSS-bone marrow derived macrophages (BMDMs) vs. respective normal and/or isotype matched IgG controls to enhance perfusion-recovery. Consistent with the bioinformatics analysis that revealed RCAN3 as a common target of miR-17 and miR-20a, Argonaute-2 pull-down assays showed decreased miR-17-20a expression and higher RCAN3 expression in the RISC complex of HSS-ECs and HSS-BMDMs vs. the respective controls. Inhibiting miR-17-20a induced RCAN3 levels to decrease ischemic angiogenesis and promoted M1-like polarization to impair perfusion recovery. Finally, using STAT3 inhibitors, S100A8/A9 silencers and VEGFR1-deficient ECs and Møs, we show that VEGF_165_b inhibition activates the miR-17-20a-RCAN3 pathway independent of VEGFR1-STAT3 or VEGFR1-S100A8/A9 in ischemic ECs and ischemic Møs, respectively.

**Conclusion:**

Our data revealed a hereunto unrecognized therapeutic ‘miR-17-20a-RCAN3’ pathway in the ischemic vasculature that is VEGFR1-STAT3/S100A8/A9 independent and is activated only upon VEGF_165_b inhibition in PAD.

## Introduction

Peripheral artery disease (PAD) occurs due to atherosclerotic occlusions in the inflow blood vessels, resulting in decreased blood flow to the lower extremities (usually legs)^1^. In its most severe form, decreased skeletal muscle perfusion results in chronic limb-threatening ischemia (CLTI), which often results in impaired wound healing and necrosis leading to limb amputation^2^. Approximately 200,000 amputations occur in the US, with PAD being the major cause^3^. Approximately 20% of patients with CLTI are also at a high risk for cardiovascular death within the first year of diagnosis^4, 5^. Currently, no approved medical therapies can revascularize the ischemic muscle and promote perfusion relief in PAD patients, indicating a greater need to identify therapies that can promote limb perfusion in PAD patients.

Vascular endothelial growth factor-A (VEGF-A) is a well-known inducer of angiogenesis^6^. Clinical trials aimed at achieving perfusion recovery in patients with PAD by activating VEGF-A-VEGFR2 signaling in PAD were not successful^2^. An inadequate understanding of VEGF-A signaling in ischemic-muscle vasculature could account for those failures. The signaling networks regulated by the VEGF-A family became more complex with the recognition of alternative splicing in the 8^th^ exon of VEGF-A that results in the formation of pro-angiogenic VEGFxxxa (xxx for the no. of amino acids. VEGF_165_a) and anti-angiogenic VEGFxxxb (VEGF_165_b) isoform families^7, 8^. The only difference between these 2 isoforms is a 6-aminoacid switch from CDKPRR (in humans and mice) in the VEGF_165_a isoform to SLTRKD (PLTGKD in mice) in the VEGF_165_b isoform.

In our recent studies, we have shown that the fraction of anti-angiogenic VEGF_165_b isoforms is ~3-fold higher than the pro-angiogenic VEGF_165_a isoforms in human PAD muscle biopsies compared to age- and sex-matched controls. In preclinical PAD models, ischemia induced ~6-fold higher numbers of VEGF_165_b^+^ ECs^9^ and ~15-fold higher VEGF_165_b^+^ macrophages^10^ (Møs) compared to nonischemic muscle. Using a highly isoform-specific monoclonal antibody raised against the 6-amino acids that neutralizes the VEGF_165_b isoform (without affecting other proangiogenic VEGF_165_a isoforms^7, 8, 9, 10, 11, 12^), we have shown that VEGF_165_b inhibition induces STAT3 activation in ischemic ECs to increase their angiogenic capacity but inhibits S1008/S1009 expression in ischemic Møs (independent of STAT3 activation) to induce a reparative M2-like phenotype^9, 10^. Furthermore, we have shown that VEGF_165_b inhibition in ischemic muscle produces therapeutic perfusion recovery in multiple preclinical PAD models^9, 10, 12,^ including eNOS-KO, Myoglobin Transgenic, and Type-2 Diabetic PAD mice^12^. However, significant gaps remain in our understanding of the genetic regulators downstream of VEGF_165_b inhibition that regulate the extent of perfusion recovery in ischemic muscle, including whether all of the effects of the antibody approach are STAT3- or S100A8/A9 dependent.

Noncoding RNAs are becoming attractive targets to treat human disease due to their ability to modulate the expression of multiple genes and biological pathways^13^. While lncRNAs are >200 base pairs in length and poorly conserved across species^14^, miRNAs are single-stranded ~16–23 nucleotide lengths that are relatively well conserved across species^15^. Despite recent advances in noncoding RNA biology, our understanding of the regulation or function of lncRNAs or miRNAs in human pathologies is still limited. In general, pairing of the miRNA seed sequence to the 3’ UTR of the target gene within a ribonucleotide binding region promotes target gene degradation or translational inhibition^16, 17^. miRNAs can occur as individuals or as a part of a miRNA cluster. For example, the miRNA-17-92 cluster (consisting of miR-17, -18, -19a, -19b, -20a, and -92a (92) members), also known as ‘OncomiR-1’, is one of the best-studied miRNA clusters due to its role in cancer biology^18, 19^. While extensive literature is available on the miR-17-92 cluster in promoting tumor progression^19, 20^, miR-92a and miR-19 within this cluster have been shown to inhibit perfusion recovery in PAD^21, 22^. Hence, we wanted to determine the role of the VEGF_165_b-miR-17-92 cluster in regulating perfusion recovery in experimental PAD.

Here, we will show that VEGF_165_b inhibition induces miR-17-20a expression in ischemic vasculature that targets Calcipressin-3 (RCAN3, shown to be an inhibitor of tumor progression^23^ and HUVEC proliferation^24^) independent of our previously published VEGFR1-STAT3 or VEGFR1-S100A8/A9 pathways to enhance perfusion recovery in experimental PAD.

## Results

### Inhibiting the antiangiogenic VEGF_165_b isoform induces truncated miR-17-20a expression (within the miR17-92 cluster) in experimental PAD models.

**Figure 1:**
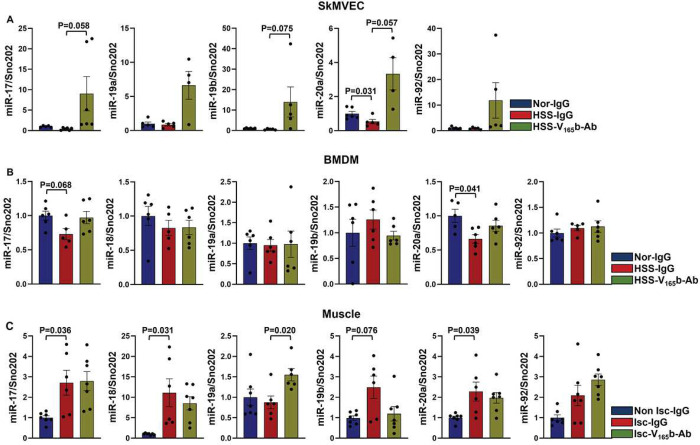
VEGF_165_b inhibition induces the expression of the miR-17-92 cluster in experimental PAD models. qPCR of miR-17-92 cluster (miR-17, miR-18, miR-19a, miR-19b, miR-20a, miR-92a) expression in A) primary skeletal muscle microvascular ECs (SkMVECs, from Balb/C mice) under normal (Nor) or hypoxic serum starvation (HSS) conditions for 24 h treated with IgG or VEGF_165_b-Ab. n=6. One-way ANOVA with Bonferroni’s multiple comparisons for miR-17, miR-19a, miR-19b, and miR-92; Brown-Forsythe ANOVA with Welch’s correction for miR-20a. C) Bone marrow-derived macrophages (BMDMs, from Balb/C mice) under Nor or HSS for 6 h treated with IgG or VEGF_165_b-Ab. n=6. One-way ANOVA with Bonferroni’s multiple comparisons for miR-17, miR-18, miR-19a, miR-19b, miR-20a, and miR-92. C) Nonischemic (Non Isc) or ischemic (Isc) Balb/CJ mouse muscle treated with IgG or VEGF_165_b-Ab at day 3 post-HLI. n=7. Brown-Forsythe ANOVA with Welch’s correction for miR-17, miR-18, and miR-20a; One-way ANOVA with Bonferroni’s multiple comparisons for miR-19a, miR-19b, and miR-92. Outliers were removed by performing the Grubbs test. P<0.05 significant. Mean±SEM.

We wanted to determine whether VEGF_165_b inhibition modulates miR-17-92 cluster expression in ischemic ECs and/or Møs. qPCR analysis of skeletal muscle microvascular ECs (SkMVECs, isolated from pooled gastrocnemius and tibialis anterior muscle) showed that hypoxia serum starvation (HSS, an *in vitro* model for PAD^25^) significantly decreased miR-20a expression but did not affect the expression of other miR-17-92 cluster members compared to normal controls. However, VEGF_165_b inhibition (10 μg/ml) induced the expression of miR-17 (P=0.058), miR-19b (P=0.075) and miR-20a (P=0.057) in HSS-SkMVECs compared to IgG (Fig-1A). Interestingly, the expression of miR-18 in SkMVECs was very low (Ct values were higher than 35, suggesting very low to no expression, data not presented). To confirm whether the lack of miR-18 expression is confined to mouse primary SkMVECs, we used HUVECs that were previously used to determine the function of VEGF_165_b in HSS conditions in our publications^9^. qPCR analysis showed a significant decrease in the expression of miR-17, miR-18, miR-19a, miR-20a, and miR-92, but not miR-19b, in HSS-HUVECs vs. normal HUVECs (Supplement-1), indicating a complete miR-17-92 cluster in human ECs. VEGF_165_b inhibition restored the expression of miR-17, miR-18, miR-19a, and miR-20a but not miR-92 to normal HUVEC levels in HSS-HUVECs vs. IgG (Supplement-1). We next examined the role of VEGF_165_b inhibition in regulating miR-17-92 cluster expression in bone marrow-derived macrophages (BMDMs). qPCR analysis showed that HSS numerically decreased the expression of miR-17 (P=0.068) and significantly decreased miR-20a expression but not the expression of other cluster members compared to normal BMDMs. Inhibiting VEGF_165_b in HSS-BMDMS restored the expression of both miR-17 and miR-20a to normal-BMDM levels (Fig-1B).

To determine whether inhibiting VEGF_165_b induces the expression of miR-17-92 cluster members (miR-17, -18, -19a, -19b, -20a, and -92) *in vivo*, we performed qPCR analysis of Balb/cJ mice (an inbred mouse strain with poor perfusion recovery post-HLI) ischemic muscle treated with IgG or VEGF_165_b-Ab (200 μg/100 μl PBS, i.m, 2 nonoverlapping sites in GA and 1 site in TA) at day 3 post HLI. qPCR analysis showed differential expression within the members of this cluster in ischemic muscle vs. nonischemic muscle. A significant increase in miR-17, miR-18, and miR-20a and a numerical increase in miR-19b (P=0.076) expression were observed in ischemic vs. nonischemic muscle, and no significant differences in miR-19a or miR-92 expression were observed in ischemic vs. nonischemic muscle. VEGF_165_b inhibition in ischemic muscle induced miR-19a expression without affecting the expression of other cluster members vs. IgG (Fig-1C). Interestingly, contrary to Balb/cJ ischemic muscle treated with VEGF_165_b-Ab (Fig-1C), VEGF_165_b inhibition in C57BL/6J mouse (an inbred mouse strain with good perfusion recovery post-HLI) ischemic muscle induced the expression of miR-17 and miR-20a with no changes in miR-19a expression vs. ischemic muscle treated with IgG (Supplement-2), suggesting that the strain of mice differentially regulates the expression of the miR-17-92 cluster to VEGF_165_b inhibition in ischemic muscle. No significant differences in other cluster members were observed between VEGF_165_b-Ab vs. IgG-treated C57BL/6J ischemic-muscle samples (Supplement-2). Based on these data, we hypothesized that VEGF_165_b inhibition induces the expression of the miR-17-20a cluster in ischemic ECs and Møs to promote perfusion recovery.

To determine whether STAT3 activation downstream of VEGF_165_b inhibition regulates miR-17-20a in ischemic ECs, we treated HUVECs and SkMVECs with a STAT3 inhibitor (S3I-201^26^) according to our previous publications^27, 28^. STAT3 inhibition did not significantly induce any changes in miR-17-20a expression in either normal- or HSS-HUVECs vs. the respective controls (Supplement-3A, B). However, while STAT3 inhibition induced miR-17-20a expression in normal SkMVECs (Supplement-3C), no significant difference in miR-17-20a expression was observed in HSS-SkMVECs (Supplement-3D). Since we have previously shown that inhibiting VEGF_165_b induces VEGFR1 activation, which activates STAT3 in HSS-ECs, we wanted to determine whether VEGFR1 regulates the miR-17-20a cluster independent of STAT3. However, no significant differences in miR-17-20a expression were observed in either normal or HSS-SkMVECs isolated from VEGFR1^+/−^ (VEGFR^−/−^ is embryonic lethal; and VEGFR1^+/−^ mice cannot upregulate VEGFR1 in response to ischemia in ischemic muscle^9^, HSS-ECs (Supplement-4) or HSS-BMDMs^10^ compared to respective VEGFR1^+/+^ controls). vs. VEGFR1^+/+^ mice (Supplement-5A, B), These data indicated that VEGF_165_b inhibition induces miR-17-20a cluster expression in HSS-ECs independent of VEGFR1-STAT3 signaling.

To determine whether S100A8/A9 plays a role in regulating miR-17-20a in ischemic Møs, we silenced S100A8 or S100A9 (Supplement-6A, B) in normal or HSS-BMDMs and examined miR-17-20a expression. Silencing S100A8 or S100A9 did not affect miR-17-20a expression in normal or HSS BMDMs (Supplement-6C, D), indicating that VEGF_165_b inhibition induces miR-17-20a expression independent of S100A8/A9 in HSS-BMDMs. Next, we wanted to determine whether VEGFR1 regulates the miR-17-20a cluster independent of S100A8/A9^10^ in HSS-BMDMs. qPCR analysis of miR-17-20a expression showed no significant difference in miR-17-20a expression in normal or HSS-challenged VEGFR1^+/−^ vs. VEGFR1^+/+^ BMDMs (Supplement-7A, B). These data indicated that inhibiting VEGF_165_b induces miR-17-20a expression in HSS-BMDMs independent of VEGFR1-S100A8/A9 signaling.

### The miR-17-20a cluster regulates perfusion recovery in PAD.

**Figure 2:**
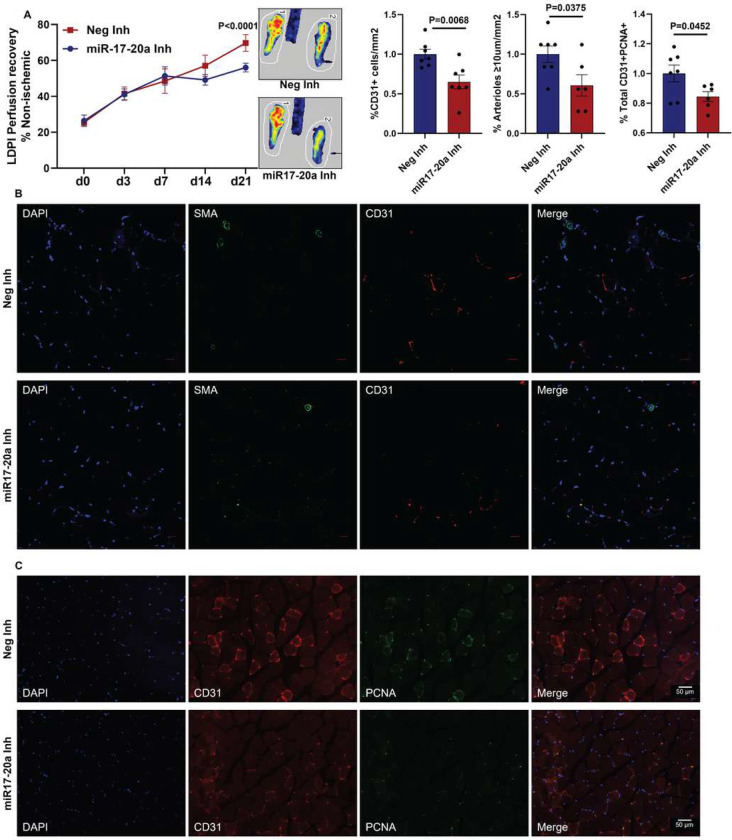
Inhibiting miR-17-20a decreases ischemic muscle revascularization in experimental PAD. A) Laser Doppler perfusion imaging of microvascular blood flow in ischemic muscle treated with a combination of miR-17, miR-20a inhibitor (miR-17-20a-Inh), or control inhibitor (Neg-Inh). n=7. Repeated measures two-way ANOVA with Bonferroni’s posttest. B) Immunohistochemical analysis of SMA (green) and CD31 (red) in ischemic gastrocnemius muscle treated with Neg-Inh or miR-17-20a-Inh at day 21 post-HLI. n=7. Unpaired T Test. Scale-50 μm. C) Immunohistochemical analysis of CD31 (red) and PCNA (green) in ischemic gastrocnemius muscle treated with Neg-Inh or miR-17-20a-Inh at day 21 post-HLI. n=7. Unpaired T Test. Outliers were removed by performing the Grubbs test. P<0.05 significant. Mean±SEM.

Since VEGF_165_b inhibition induced miR-17-20a expression in C57BL/6J ischemic muscle (Supplement-2), we hypothesized that the ability of C57BL/6J ischemic muscle to induce miR-17-20a expression upon VEGF_165_b inhibition enhances perfusion recovery in experimental PAD. To test this hypothesis, we inhibited miR-17 and miR-20a in C57BL/6J skeletal muscle by i.m. delivery of miR-17+miR-20a antagomirs (100 μM miR-17 and miR-20a antagomir or equimolar concentration of nontargeting antagomir (inhibitor) in 100 μl of PBS injected at 2 nonoverlapping sites in GA and 1 site in TA) at days 0, 3, 7, 14, and 21 post-HLI^9, 10, 29^ (Supplement-8). miR-17-20a antagomirs significantly decreased perfusion recovery in C57BL/6J ischemic muscle vs. control inhibitor (day 21 post-HLI: control inhibitor-69.69±1.9 vs. miR-17-20a inhibitor-56.04±0.9, P<0.0001, Fig-2A). Immunohistochemistry of CD31 and α-smooth muscle actin (SMA, ≥10 μm vessels) showed a significant decrease (1.5-fold) in EC numbers and SMA^+^ arterioles in C57BL/6J ischemic muscle treated with miR-17-20a inhibitors vs. control inhibitors at day 21 post-HLI (Fig-2B). Consistent with lower EC numbers, immunohistochemical analysis revealed a significant decrease in the fraction of proliferating ECs (PCNA^+^CD31^+^ in total PCNA^+^ cells) in miR-17-20a inhibitor-treated C57BL/6J ischemic muscle vs. control inhibitor at day 21 post-HLI (Fig-2C).

**Figure 3:**
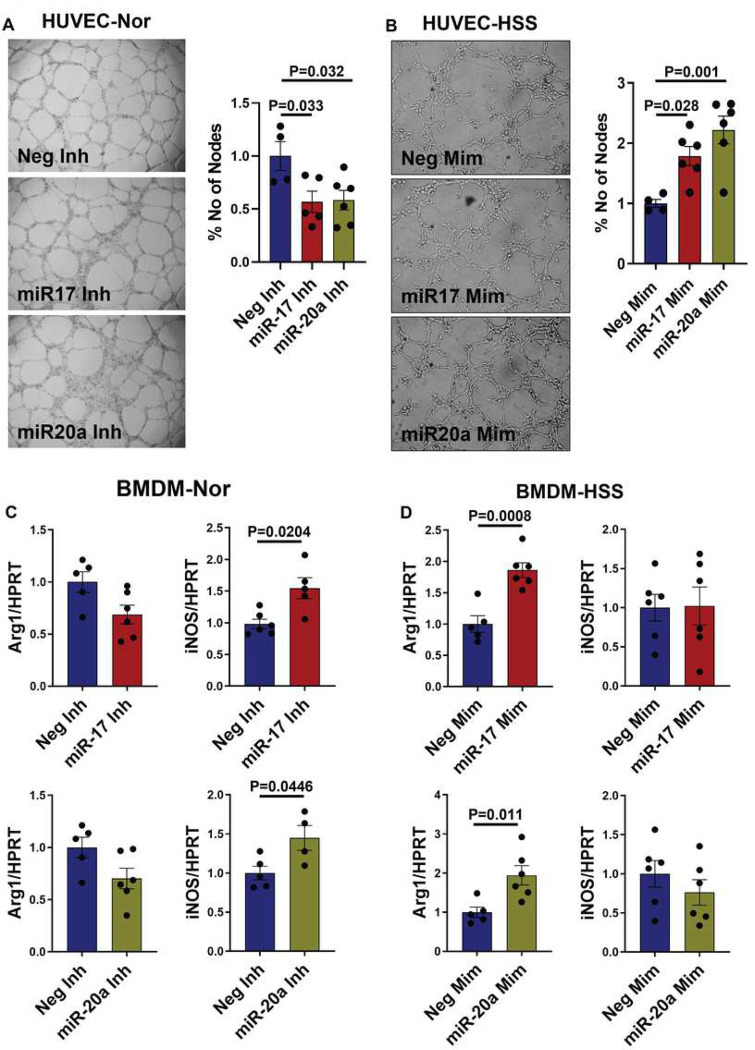
miR-17-20a induces ischemic angiogenesis and an M2-like macrophage phenotype *in vitro*. A) *In vitro* tube formation assay of normal HUVECs transfected with negative inhibitor (Neg Inh, n=4), miR-17 inhibitor (miR-17 Inh, n=5) or miR-20a inhibitor (miR-20a Inh, n=6) on growth factor-reduced Matrigel (GFRM). One-way ANOVA with Dunnett’s posttest. B) *In vitro* tube formation assay of HSS-HUVECs transfected with Neg-Mim, miR-17-Mim, or miR-20a-Mim on GFRM. n=6. One-way ANOVA with Dunnett’s posttest. C) qPCR of arginase-1 (Arg1) and inducible nitric oxide synthase (iNOS) expression in normal BMDMs transfected with negative inhibitor (Neg Inh), miR-17 inhibitor (miR-17 Inh) or miR-20a inhibitor (miR-20a Inh). n=6. Unpaired T Test. D) qPCR analysis of Arg1 and iNOS expression in HSS BMDMs transfected with Neg-Mim, miR-17-Mim, or miR-20a-Mim. n=6. Unpaired T Test. P<0.05 significant. Mean±SEM.

We next wanted to determine whether the increased angiogenic capacity in ischemic ECs post VEGF_165_b inhibition is mediated by increased miR-17-20 expression. We performed gain-of-function and loss-of-function experiments by inhibiting miR-17 and miR-20a in normal HUVECs (Supplement-9A) and overexpressing miR-17 and miR-20a in HSS-HUVECs (Supplement-9B). While normal HUVECs transfected with miR-17 or miR-20a antagomirs showed a significant decrease in tube-like formation on growth factor-reduced Matrigel (GFRM, control inhibitor: 129.5±17.53, miR-17 inhibitor: 73.6±12.9, miR-20a inhibitor: 75.67±11.9, P<0.05, Fig-3A), HSS-HUVECs transfected with miR-17 or miR-20a mimics showed a significant increase in tube-like structures on GFRM (control mimic: 83.75±5.7, miR-17-mimic: 149.5±13.4, miR-20a mimic: 185.8±19.2, P<0.05, Fig-3B) vs. the respective controls, indicating that the miR-17-20a cluster induces ischemic EC angiogenic capacity.

Since VEGF_165_b inhibition induces an M2-like phenotype in ischemic Møs, we next wanted to determine the role of miR-17-20a in regulating ischemic Mø polarization^10, 29^. Gain-of-function and loss-of-function experiments were performed by inhibiting miR-17 and miR-20a in normal BMDMs (Supplement-10A) and overexpressing miR-17 and miR-20a in HSS BMDMs (Supplement-10B). qPCR analysis showed no significant differences in Arg1 expression (M2 marker) but a significant increase in iNOS expression (M1 marker) in normal BMDMs transfected with miR-17 or miR-20a antagomirs (Fig-3C). In HSS-BMDMs transfected with miR-17 or miR-20a mimics, a significant increase in Arg-1 expression without any changes in iNOS expression was observed (Fig-3D). These data indicated that the miR-17-20a cluster induces an M2-like-reparative phenotype in ischemic Møs.

### miR-17-20a targets RCAN3 in ischemic vasculature.

**Figure 4:**
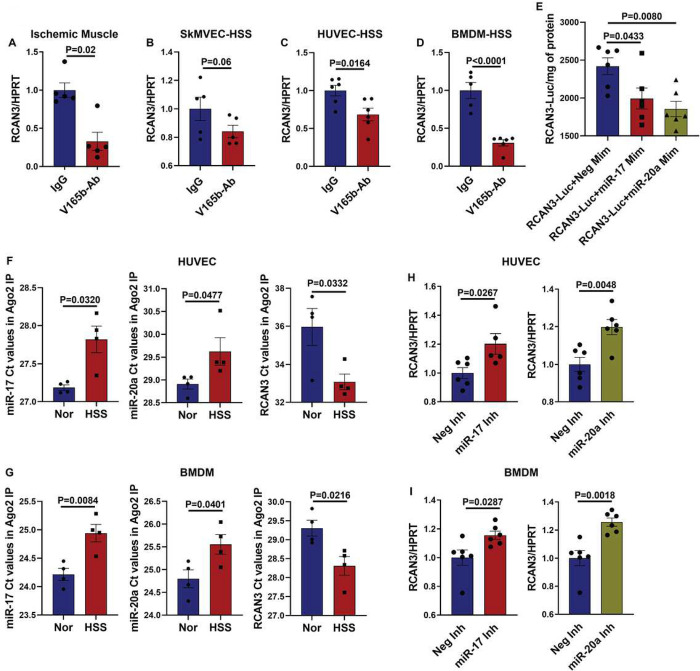
miR-17-20a targets RCAN3 in ischemic endothelial cells and macrophages. qPCR of RCAN3 expression in A) IgG- or V_165_b-Ab-treated ischemic muscle. n=5. Unpaired T Test. B) IgG- or V_165_b-Ab-treated HSS-SkMVECs. n=6. Unpaired T Test. C) IgG- or V_165_b-Ab-treated HSS-HUVECs. n=6. Unpaired T Test. D) IgG- or V_165_b-Ab-treated HSS-BMDMs. n=7. Unpaired T Test. P<0.05 significant. E) 3’ UTR luciferase assay in HEK293 cells transfected with RCAN3 3’UTR luciferase plasmid followed by transfection with Neg-Mim, miR-17-Mim, or miR-20a-Mim. n=6. One-way ANOVA with Dunnett’s posttest. F) qPCR analysis of miR-17 (unpaired t test with Welch’s correction), miR-20a, and RCAN3 Ct values in Ago2-IP fractions from normal and HSS HUVECs. n=4. Unpaired T test. G) qPCR analysis (Ct values) of miR-17, miR-20a, and RCAN3 expression in RCAN3-IP fractions from normal and HSS BMDMs. n=4. Unpaired T Test. H, I) qPCR of RCAN3-expression in H) HSS-HUVECs and I) HSS-BMDMs transfected with negative inhibitor (Neg-Inh), miR-17 inhibitor (miR-17-Inh) or miR-20a inhibitor (miR-20a Inh). n=6. Unpaired T Test. Outliers were removed by performing the Grubbs test. P<0.05 significant. Mean±SEM.

Since miRNA binding to their target genes results in transcriptional or translational inhibition^16^, we next wanted to determine the gene target of miR-17 and/or miR-20a that regulates perfusion recovery post-VEGF_165_b inhibition in ischemic muscle. Based on the bioinformatics analysis or miR-17 and miR-20a predicted targets using the mirdb target prediction database^30, 31^ and Targetscan7.2^32^ that identified RCAN3 as a potential common target of miR-17 and miR-20a, we first examined whether VEGF_165_b inhibition modulates RCAN3 expression in experimental PAD models. qPCR analysis showed a significant decrease in RCAN3 expression in ischemic muscle (Fig-4A), HSS-ECs (Fig-4B), HSS-HUVECs (Fig-4C) and HSS-BMDMs (Fig-4D) treated with VEGF_165_b-Ab vs. IgG, indicating that VEGF_165_b inhibition decreases RCAN3 expression in experimental PAD models.

To obtain direct evidence of miR-17 and miR-20a targeting RCAN3, we performed a RCAN3-3’ UTR luciferase assay by transfecting HEK293 cells with RCAN3-3’UTR-Luc followed by transfection with miR-17 or miR-20a mimics. A luciferase assay showed a significant decrease in luciferase activity in miR-17- and miR-20a-transfected RCAN3-3’UTR-Luc HEK293 cells, indicating that miR-17 and miR-20a bind and inhibit RCAN3 expression (Fig-4E). Furthermore, Argonaute-2 immunoprecipitated complexes in normal vs. HSS-HUVECs (Supplement-11A) and normal vs. HSS-BMDMs (Supplement-11B) showed a significant decrease in RCAN3 Ct values (indicating higher expression) and significantly higher miR-17 and miR-20a Ct values (indicating lower expression) in HSS-HUVECs (Fig-4F) and HSS-BMDMs (Fig-4G) vs. respective normal controls. Consistent with these findings, qPCR analysis of HSS-HUVECs transfected with miR-17 or miR-20a antagomirs showed a significant increase in RCAN3 expression vs. control antagomir (Fig-4H) and a significant increase in RCAN3 expression in HSS-BMDMs transfected with miR-17 or miR-20a antagomir vs. control inhibitor (Fig-4I). These data indicated that VEGF_165_b inhibition induces miR-17-20a expression in the ischemic vasculature, which inhibits RCAN3 expression to promote perfusion recovery in PAD.

### RCAN3 regulates ischemic endothelial angiogenic capacity and macrophage polarization.

**Figure 5:**
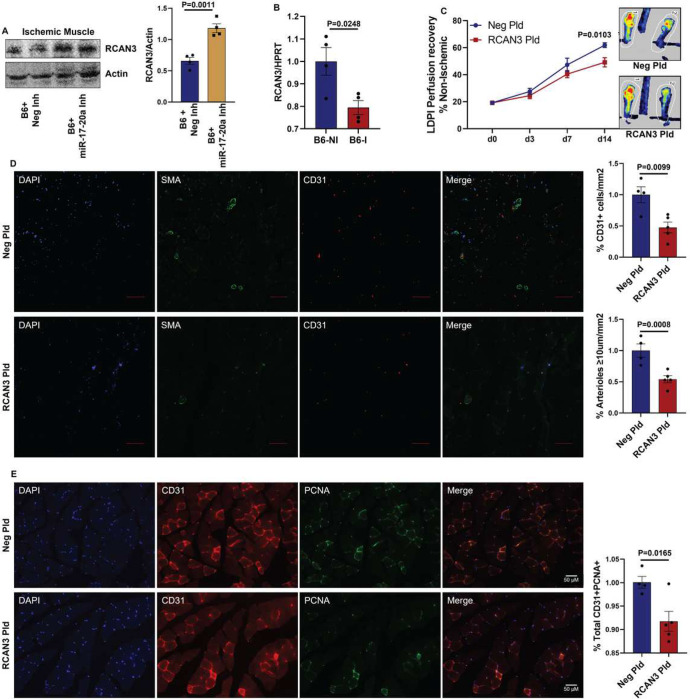
RCAN3 inhibits perfusion recovery in experimental PAD. A) Western blot analysis of RCAN3 in ischemic muscle treated with Neg Inh or a combination of miR-17 and miR-20a inhibitor (miR-17-20 Inh). n=4. Unpaired T Test. B) qPCR of RCAN3 expression in C57BL/6J nonischemic (NI) and ischemic (I) muscle. n=4. Unpaired T test. C) Laser Doppler perfusion imaging of microvascular blood flow in ischemic muscle transfected with a control plasmid (Neg Pld, n=4) or RCAN3-expressing plasmid (RCAN3 Pld, n=5). Repeated measures two-way ANOVA with Bonferroni’s posttest. D) Immunohistochemical analysis of SMA (green) and CD31 (red) in ischemic gastrocnemius muscle treated with Neg Pld (n=4) or RCAN3 Pld (n=5) at day 14 post-HLI. Unpaired T Test. E) Immunohistochemical analysis of CD31 (red) and PCNA (green) in ischemic gastrocnemius muscle treated with Neg Pld (n=4) or RCAN3 Pld (n=5) at day 14 post-HLI. Unpaired T Test. P<0.05 significant. Mean±SEM.

Limited information exists on the role of RCAN3 in regulating angiogenesis in general in PAD. First, we determined an *in vivo* role of miR-17-20a in regulating RCAN3 in preclinical PAD. Since C57BL/6 can upregulate miR-17-20a expression in ischemic muscle (Supplement-2), we treated ischemic muscle with control miR-17-20a inhibitors (100 μM, i.m. 3 sites in muscle (2 in GA and 1 in TA) at day 0 and examined RCAN3 expression at day 3 post HLI. qPCR analysis showed that miR-17-20a inhibition in C57BL/6J ischemic muscle significantly induced RCAN3 levels compared to the negative inhibitor at day 3 post-HLI (Fig-5A). Subsequent qPCR analysis showed a significant decrease in RCAN3 expression in C57BL/6J ischemic muscle vs. nonischemic muscle (Fig-5B). Taken together, these data indicated that the ability of C57BL/6 to induce miR-17-20a expression in ischemic muscle targets RCAN3 to promote perfusion recovery.

To test the role of RCAN3 in regulating PAD, we induced RCAN3 expression by electroporating a RCAN3-expressing plasmid (vs. control plasmid, Supplement-12) into C57BL/6J skeletal muscle (GA and TA) and performed HLI. Laser Doppler showed a significant decrease in perfusion recovery in C57BL/6J mouse ischemic muscle treated with RCAN3-expressing plasmid vs. control plasmid (day 14: control plasmid 61.9±1.74 vs. RCAN3 plasmid 49.8±3.4), indicating that increased RCAN3 levels impair perfusion recovery in PAD (Fig. 5C). Immunohistochemical analysis of CD31 and SMA (≥10 μm vessels) showed a significant decrease (~2-fold) in EC numbers and SMA+ arterioles in the ischemic muscle transfected with RCAN3-expressing plasmid vs. control at day 14 post-HLI (Fig-5D). Consistent with the lower EC numbers, RCAN3 overexpression decreased the fraction of proliferating ECs (PCNA^+^CD31^+^ in total PCNA^+^ cells) vs. control plasmid at day 14 post-HLI (Fig-5E).

**Figure 6:**
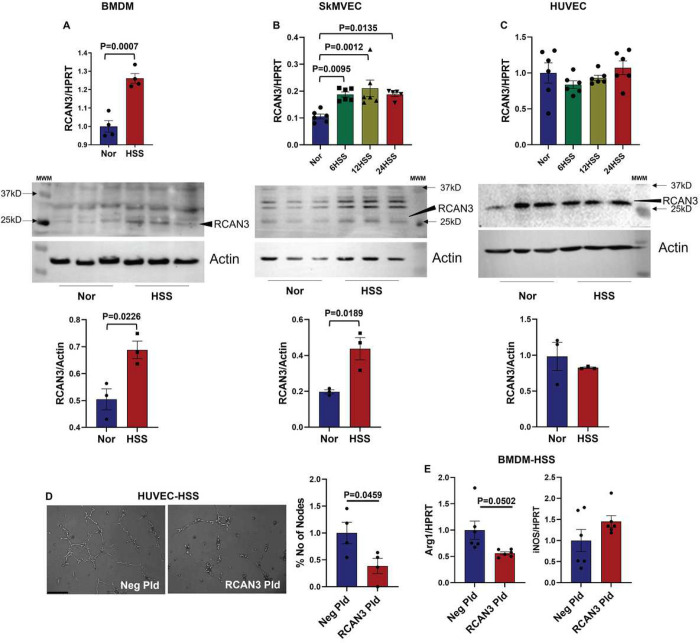
RCAN3 inhibits ischemic angiogenesis and induces an M1-like macrophage phenotype *in vitro*. qPCR analysis (top panel) of RCAN3 in A) BMDMs (normal n=4, HSS n=4), unpaired t test, B) SkMVECs (normal n=6, 6 h HSS n=6/time point), one-way ANOVA with Dunnett’s posttest, and C) HUVECs (normal n=6, HSS n=6/time point), one-way ANOVA with Dunnett’s posttest. Western blot analysis (lower panel) of RCAN3 in normal vs. HSS-challenged A) BMDMs (n=3), B) SkMVECs, (n=3) and C) HUVECs (n=3). Unpaired T test. D) *In vitro* tube formation assay of HSS-HUVECs transfected with Neg Pld or RCAN3 plasmid on GFRM. n=4. Unpaired T Test. E) qPCR of arginase-1 (Arg1, (unpaired t test with Welch’s correction)) and inducible nitric oxide synthase (iNOS) expression in HSS-BMDMs transfected with Neg Pld or RCAN3 Pld, n=6. Unpaired T Test. P<0.05 significant. Mean±SEM.

We next wanted to determine the cell-specific function of RCAN3 in regulating ischemic-EC angiogenic capacity and ischemic-Mø polarization. qPCR and western blot analysis showed a significant increase in RCAN3 levels in HSS-BMDMs (Fig-6A) and HSS-SkMVECs (Fig-6B) vs. the respective normal controls. No significant difference was observed between normal vs. HSS-HUVECs (Fig-6C). However, functionally, overexpressing RCAN3 (Supplement-13A) significantly decreased HSS-EC tube-like formation on GFRM vs. control plasmid (Fig-6D). RCAN3 overexpression (Supplement-13B) significantly decreased Arg-1 expression without changing iNOS expression^10, 29^ in HSS-BMDMs, indicating an induction of the M1-like phenotype (Fig-6E). These data indicated that RCAN3 inhibits ischemic EC angiogenic capacity and induces an M1-like cytotoxic phenotype to impair perfusion recovery in PAD.

Finally, we wanted to determine whether VEGFR1-STAT3 or VEGFR1-S100A8/A9 signaling regulates RCAN3 expression in ischemic ECs or ischemic Møs. STAT3 inhibition did not induce any significant changes in RCAN3 expression in normal or HSS-HUVECs (Supplement-14A) or in normal or HSS-SkMVECs (Supplement-14B). No significant differences in RCAN3 expression were observed in HSS-SkMVECs from VEGFR1^+/−^ vs. VEGFR1^+/+^ mice (Supplement-14C). Furthermore, silencing S100A8/A9 did not affect RCAN3 expression in normal HSS-BMDMs (Supplement-15A, B). No significant differences in RCAN3 expression were observed in HSS-BMDMs from VEGFR1^+/−^ vs. VEGFR1^+/+^ mice (Supplement-15C). These data indicated that inhibiting VEGF_165_b decreases RCAN3 expression in HSS-ECs and HSS-BMDMs independent of VEGFR1-STAT3 or VEGFR1-S100A8/A9 signaling, respectively.

## Discussion

In our efforts to understand the mechanisms involved in regulating the angiogenic response to hind limb ischemia in general and specifically for advancing our understanding of the VEGF_165_b isoform, our study presents a novel miR-17-20a-RCAN3 pathway that occurs specifically with the depletion of the antiangiogenic VEGF_165_b in PAD. The ability of skeletal muscle to recover from ischemic damage that occurs experimentally from HLI and repeatedly during daily activity in patients with PAD is directly dependent on the extent of the angiogenic response in ischemic muscle^2^. To the best of our knowledge, our current study is the first to demonstrate a pathway by which removing the inhibitory effect of VEGF_165_b in ischemic muscle allows the expression of a truncated miR-17-20a cluster in ischemic ECs and Møs that targets RCAN3 to revascularize ischemic muscle in experimental PADs. The miR-17-92 cluster is a highly studied miRNA cluster in cancer research^19, 20, 22^. While studies, in general, are focused on the whole miR-17-92 cluster, in our study, we present specific roles of miR-17 and miR-20a within the miR-17-92 cluster that are regulated by VEGF_165_b inhibition. Although the first evidence of an oncogenic role (by promoting cell survival, proliferation, and angiogenesis) in B-cell lymphoma involved a truncated miR-17-92 cluster (without miR-92), hence the name ‘OncomiR’^18, 33, 34^ was given, two paralogs of the miR-17-92 cluster are already known to occur in humans: miR106b-25 (miR-106b, -93, -25) and miR-106a-363 (miR-106a, -18b, -19b-2, -20b, -92a-2, and -363) clusters^35^.

The 15 miRNAs from these 3 paralogous clusters form four seed families that include the miR-17 family (miR-17, miR-20a, miR-106a, miR-20b, miR-106b, and miR-93); miR-18 family (miR-18a, miR-18b); miR-19 family (miR-19a, miR-19b-1, miR-19b-2) and miR-92 family (miR-92a, miR-92-a-2, miR-363 and miR-25). A previous study by Hazarika et al^25^ from our group showed that miR-106b, miR-93, miR-106a, and miR-17 are among the top 10 differentially regulated genes between C57BL/6J vs. Balb/cJ ischemic muscle at day 3 post-HLI. Consistent with these data, in our current study, we observed miR-17 to be one of the significantly different miRNAs in the ischemic muscle of C57BL/6J and Balb/cJ mouse strains with proangiogenic properties in the ischemic environment. Our previous studies have also shown that miR-93 within the miR-106b-25 cluster exhibits pro-angiogenic properties in an ischemic environment^25, 29^, and miR-106a within the miR-106a-363 cluster induces ischemic angiogenesis (unpublished data). Taken together, these studies further indicate that miRNAs within the miR-17 seed sequence family may play critical roles in regulating the pathology of PAD.

Limited information is available on the role of the miR-17-92 cluster members in cardiovascular diseases, including PAD. Hinkel et al^21^ showed that inhibiting miR-92a improved functional recovery in PAD^21^. Landskroner-Eiger et al^22^ have shown improved limb arteriogenesis in EC-specific miR-17-92 cluster-deficient mice in experimental PAD, and this study shows that specific targeting of Frizzled Class Receptor 4 (FZD4) and LDL Receptor Related Protein 6 (LRP6) by miR-19 within the cluster plays a causal role in decreasing blood flow recovery^22^. In contrast, a recent report by Chamorro-Jorganes et al^36^. showed that EC-specific miR-17-92-deficient mice have blunted physiological retinal angiogenesis as well as diminished VEGF-induced angiogenesis^36^. Furthermore, deletion of the miR-17-92 cluster in renal proximal tubules or in ECs resulted in severe renal dysfunction and promoted microvascular rarefaction^37, 38^. In our current study, increased expression of the miR-17-20a cluster by VEGF_165_b inhibition enhanced perfusion recovery. These data indicate that the phenotypic effect observed in gene knockout models is not the same as fine-tuning^39^ a miRNA level/function. This can be reflected in the lack of angiogenesis in miR-17-92 gene knockout models^22^ vs. increased ischemic-muscle revascularization when the miR-17-20a levels/function are fine-tuned (similar to their expression in normal conditions) by VEGF_165_b inhibition. Taken together, these data indicate that VEGF_165_b inhibition fine-tunes the expression of the miR-17-20a cluster, which is sufficient to induce ischemic-muscle revascularization in PAD.

Interestingly, Chamorro-Jorganes et al^36^ described that VEGF induces miR-17-92 expression via ELK1 (extracellular signal-regulated kinase (ERK)-ETS-like-1), suggesting a potential role of VEGFR2 signaling in regulating the miR-17-92 cluster^36^. However, our recent reports have shown that VEGF_165_b induces VEGFR2 activation and that inhibiting VEGF_165_b decreases VEGFR2 activation. We anticipated that VEGF_165_b inhibition will induce the miR17-20a-RCAN3 pathway dependent on VEGFR1-STAT3 (in ischemic ECs) or VEGFR1-S100A8/A9 (in ischemic Møs). However, our data showed that inhibiting VEGF_165_b regulates the miR-17-20-RCAN3 pathway independent of VEGFR1-STAT3 or VEGFR1-S100A8/A9 signaling in ischemic ECs and ischemic Møs, respectively. While a full explanation of the involvement of alternative signaling pathways regulated by VEGF_165_b to control miR-17-20a expression is beyond the scope of this report, these data suggest the possibility of direct epigenetic regulation by VEGF_165_b isoforms in ischemic vasculature. In support of this, previous reports have shown that VEGF-A accumulates in the nucleus during wound healing and in response to hypoxia^40, 41^. Since VEGF_165_a and VEGF_165_b isoforms only differ in exon 8, Further studies to determine the role of VEGF_165_b in nuclear vs. membrane compartments will present evidence for novel epigenetic vs. receptor-mediated signaling mechanisms downstream of VEGF_165_b inhibition that regulate miR-17-20a cluster expression in ways different from VEGFR2 or our previously published VEGFR1 signaling in ischemic vasculature.

Distinct functions and expression of the specific miRs within this cluster have been well established in several tumor studies^42, 43, 44, 45, 46, 47, 48, 49, 50, 51^. Reports on the 6 miRs that comprise the miR-17-92 cluster have reported differential expression patterns. For example, miR-92 is expressed at much higher levels than the rest of the cluster members in several tumors, including glioma, colorectal cancer, and breast cancer^42, 45, 46^. Accordingly, in our current study, we consistently observed lower miR-18 expression than other cluster members in primary skeletal muscle, ECs, and BMDMs under normal or ischemic conditions. Although the miR-17-92 cluster is conserved between humans and mice, it is important to note a relatively lower expression (or lack of) of miR-18 expression in primary mouse ECs but not in human ECs in our study. This suggests a more complex evolutionary regulation of this cluster across species, and distinct context-dependent mechanisms operate in biogenesis, processing, and/or degradation within the members of this miRNA cluster^52, 53^.

Our current study identified RCAN3 as a novel regulator of PAD. While HSS induced RCAN3 expression in SkMVECs, no significant difference in RCAN3 expression was observed in HSS-HUVECs vs. normoxic controls. Nevertheless, RCAN3 overexpression inhibited HUVEC angiogenic capacity, and silencing RCAN3 enhanced SkMVEC angiogenic capacity, indicating a functional role of the miR-17-20a-RCAN3 pathway in regulating ischemic angiogenesis. Limited information is available on the pathological roles of RCAN3. Recent studies have shown that RCAN3 decreases arthritis development in collagen-induced murine models^54,^ and overexpressing RCAN3 or RCAN3-derived peptide has been shown to inhibit tumor progression^23^. Furthermore, RCAN3 has been shown to induce antiproliferative effects in HUVECs^24^. Our data showing that transcriptional and translational repression of RCAN3 by miR-17 and miR-20a enhances perfusion recovery in preclinical PAD models presents RCAN3 as a putative miR-17 and miR-20a target that regulates perfusion recovery in PAD. While further studies are needed to determine whether sex plays a role in regulating the miR-17-20a-RCAN3 pathway in experimental PAD, our study is the first report identifying RCAN3 as a downstream regulator of VEGF_165_b. The ability of RCAN3 to regulate ischemic EC angiogenic capacity and Mø polarization makes it an attractive PAD therapeutic.

## Conclusion.

We present new evidence that removal of VEGF_165_b is necessary to induce miR-17 and miR-20a expression that revascularizes ischemic muscle by targeting RCAN3, a novel regulator of PAD. Further studies are needed to understand the molecular mechanisms that regulate the transcriptional control of miR17-92 cluster members that results in their distinct expression pattern post VEGF_165_b inhibition. Advances in miRNA therapeutics and monoclonal antibody-based therapeutics point toward the potential to target miR-17-20a and RCAN3 for clinical applications.

## Data Availability

All data generated or analyzed during this study are included in this published article and its supplementary information files.

## Key Points

- VEGF_165_b inhibits a truncated miR17-20a cluster in ischemic endothelial cells and macrophages to inhibit the ischemic endothelial angiogenic capacity and induce an M1-like phenotype, respectively.
- miR-17-20 targets RCAN3 to induce the angiogenic capacity of hypoxic endothelial cells and promote an M2-like reparative phenotype in hypoxic macrophages, resulting in enhanced perfusion recovery in experimental PAD.
- RCAN3 inhibits the angiogenic capacity of hypoxic endothelial cells and drives an M1-like cytotoxic macrophage phenotype in hypoxic macrophages.
- Inhibiting VEGF_165_b is necessary to increase the expression of the miR-17-20a cluster that targets RCAN3 for transcriptional and translational inhibition.
